# FPGA-based CCD signal acquisition and transmission system design

**DOI:** 10.1038/s41598-024-52438-0

**Published:** 2024-01-22

**Authors:** Xuxiang Peng, Yun Tang, Jingfeng Li, Penghui Zou, Ping Liao, Jiatao Yan, Meng Shen, Ling Zhou

**Affiliations:** 1 Hunan Province Key Laboratory of Intelligent Sensors and Advanced Sensors Materials, School of Physics and Electronic Science, Hunan University of Science and Technology, Xiangtan, 411201 Hunan China; 2 Wuhan National Laboratory for Optoelectronics (WNLO), Huazhong University of Science and Technology, Wuhan, 430074 Hubei China; 3 School of Intelligent Manufacturing, Hunan University of Science and Engineering, Yongzhou, 425199 China

**Keywords:** Electrical and electronic engineering, Applied optics

## Abstract

In order to facilitate the analysis and processing of optical signals, an FPGA-based CCD signal acquisition and data transmission system is designed in this work. The system uses an FPGA as the main control device, the TCD1304DG/AP chip as the optical signal detector, and the CYUSB3KIT-003 development board product by Cypress for data transmission. Verilog and Python languages are employed for modular design and on-board verification. Through the coordination of each module, the system successfully achieves CCD signal data acquisition and transmission.

## Introduction

The spectrometer is an optical device that employs the principle of light dispersion to decompose the complex compositions of light to form a spectrum. It finds applications in various fields of detection^1^. Traditional detection techniques such as spark direct reading specroscopy, inductively coupled plasma emission spectroscopy, and chemical titration, among others, have limitations that are difficult to overcome, making it hard to meet the increasing demands for detection^2^. LIBS technology utilizes laser ablation on the surface of the analyzed sample, and then analyzes the plasma emission spectra to determine the composition and content of the material^3^. This technique offers the advantage of analyzing samples without preparation^4^. Meanwhile, it enables the detection of almost all elements and the simultaneous analysis of multiple elements, significantly enhancing detection efficiency^5^. As a result, LIBS is applied to some industries that require high precision and high efficiency detection, including metallurgy, prospecting and mining, aerospace, and environmental monitoring^6,7^. However, traditional spectrometers which are used in LIBS technology have certain limitations that are difficult to make up, including large size, unsuitability for harsh environments, complex structure, and high cost, which unable to achieve real-time detection and the usage in complex environments^8,9^. With the development of optics and the advent of CCD detectors, it is characterized by high efficiency, affordability, and ease of controls^10,11^. Therefore, the spectrometer is developing in the director of small portable while maintaining high sensitivity and time-resolved capabilities in the future, holding great promise^12–14^.

This work will focus on the control, signal acquisition, and transmission of the CCD detector and combine these aspects with the contemporary and high-speed transmission modular design to propose a practical solution for achieving spectrometer miniaturization while preserving a high time-resolution capability^15^. The acquisition and transmission system presented in this paper is distinguished by its versatility, high resolution, and rapid data transmission.

## Principle of spectrometer

**Figure 1:**
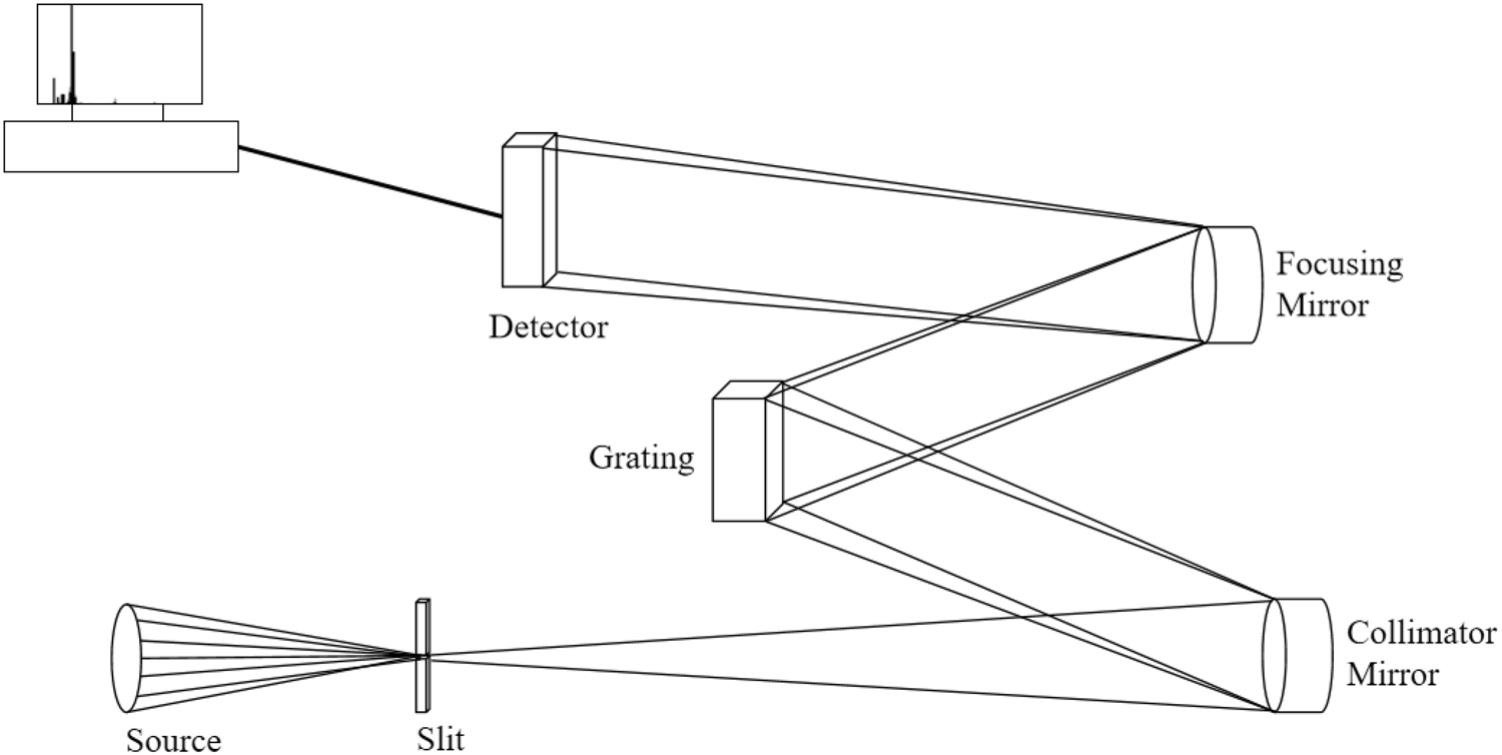
C-T spectrometer.

The classical spectrometer system, known as the Czerny-Turner spectrometer or C-T structure spectrometer, is depicted in Fig. 1. This spectrometer typically comprises some components such as an optical fiber, incident slit, collimating objective, diffraction grating, converging objective, sensor, and other elements^16^. Among these components, the most important one is sensor which converts the optical signal into the corresponding electrical signal. Subsequently, the measured spectral data is analyzed through the data processing module of the host computer to derive the composition of the detection target.

## Hardware circuit design

**Figure 2:**
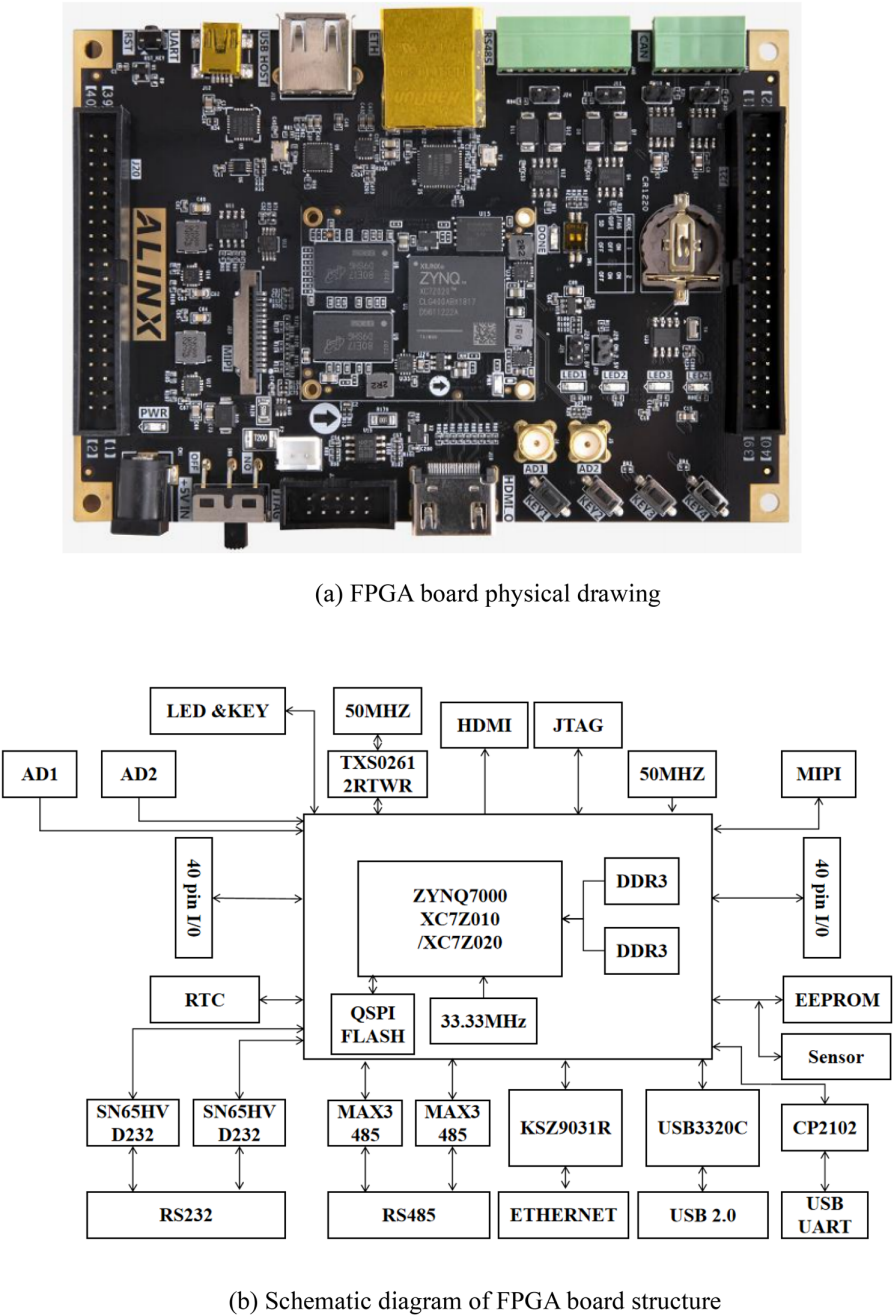
(**a**) FPGA board physical drawing. (**b**) Schematic diagram of FPGA board structure.

In this design, the FPGA is chosen as the primary controller, and the hardware circuit module consists of the following components: FPGA control chip, CCD detector, signal acquisition circuit, signal conversion circuit, and data transmission circuit^17^. The overall system operating rate relies on the maximum operating rate of the hardware circuit. The hardware circuit has specific requirements for the spectrometer’s sampling rate, data transmission, and analysis rate. For the control chip selection, the ALINX AXC7Z010 development board kit is utilized, which features XILINX’s Zynq7000 SOC chip solution. This core board offers forty programmable IO ports and two external ADC signal input interfaces. Additionally, the PL logic end is equipped with a 50 MHz crystal oscillator, supporting output stabilization in up to eight times frequency to meet the maximum frequency demands of both external control devices and internal operations. The physical and structural schematic diagrams of the design are shown below, with Fig. 2a displaying the physical diagram and Fig. 2b illustrating the structural diagram. ALINX AXC7Z010 has 1 Gigabit Ethernet Interface, 1 USB2.0 HOST Interface, 1 HDMI Output Interface, 1 SD Card Interface, 1 UART USB Interface, 1 SD Card Interface, 1 SD Card Interface interface, 1 HDMI output interface, 1 SD Card interface, 1 UART USB interface, 1 SD Card interface, 1 MIPI interface, 2 CAN bus interfaces, 2 RS485 bus interfaces, 2 AD input interfaces, 2 40-pin expansion ports, and a number of buttons and LEDs.

### Signal acquisition circuit

The signal acquisition circuit is a crucial component of the spectrometer system, responsible for converting optical signals into electrical signals. The main content of its design is the selection and application of the CCD detector. For the CCD detector selection, the TCD1304DG is chosen. The TCD1304DG is a line array CCD sensor with 3648 sampling pixels.

**Figure 3:**
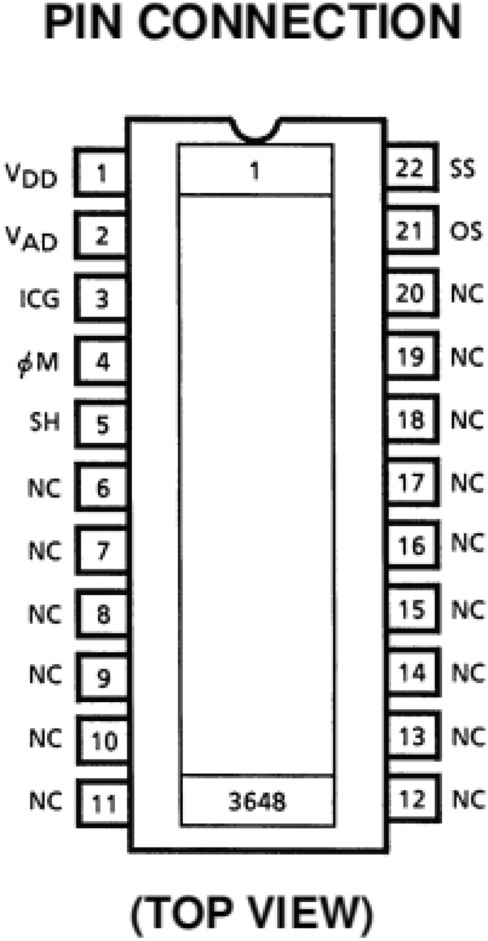
TCD1304DG circuit diagram (taken from the datasheet for the TCD1304DG (page 1)).

The circuit diagram of the TCD1304DG is depicted in Fig. 3, featuring a total of 22 pins. Among them, ${V}_{\varphi M}$ serves as the main clock signal, ${V}_{SH}$ represents the frame transfer signal, ${V}_{ICG}$ denotes the reset signal, and ${V}_{OS}$ corresponds to the analog voltage value output signal. The TCD1304DG is operated by controlling the input of the ${V}_{SH}$, ${V}_{ICG}$, and ${V}_{\varphi M}$ signals. Subsequently, the analog voltage value is outputted through ${V}_{OS}$ after undergoing analog-to-digital conversion in the AD conversion circuit within the signal processing module. The resulting data information is then processed by the FPGA. In summary, the TCD1304DG circuit diagram, shown in Fig. 3, encompasses 22 pins, with ${V}_{\varphi M}$ as the main clock signal, ${V}_{SH}$ as the frame transfer signal, ${V}_{ICG}$ as the reset signal, and ${V}_{OS}$ as the analog voltage value output signal. By manipulating the input of ${V}_{SH}$, ${V}_{ICG}$, and ${V}_{\varphi M}$ signals, the TCD1304DG facilitates the output of analog voltage values via ${V}_{OS}$. These values are internally and subsequently subjected to analog-to-digital conversion by the AD conversion circuit in the signal processing module, following by which the data information is handed over to the FPGA for further processing.

### Signal processing circuit

The output signal from the CCD detector needs to be converted from analog to digital before it can be processed by the FPGA. This is because the FPGA requires digital input signals, while the CCD detector produces analog signals. In this design, the AX7Z010 development board which features a four-way AD input port is utilized. Two of these ports are dedicated to external analog signal acquisition and conversion, while the other two are used for measuring various parameters within the development board.

**Figure 4:**
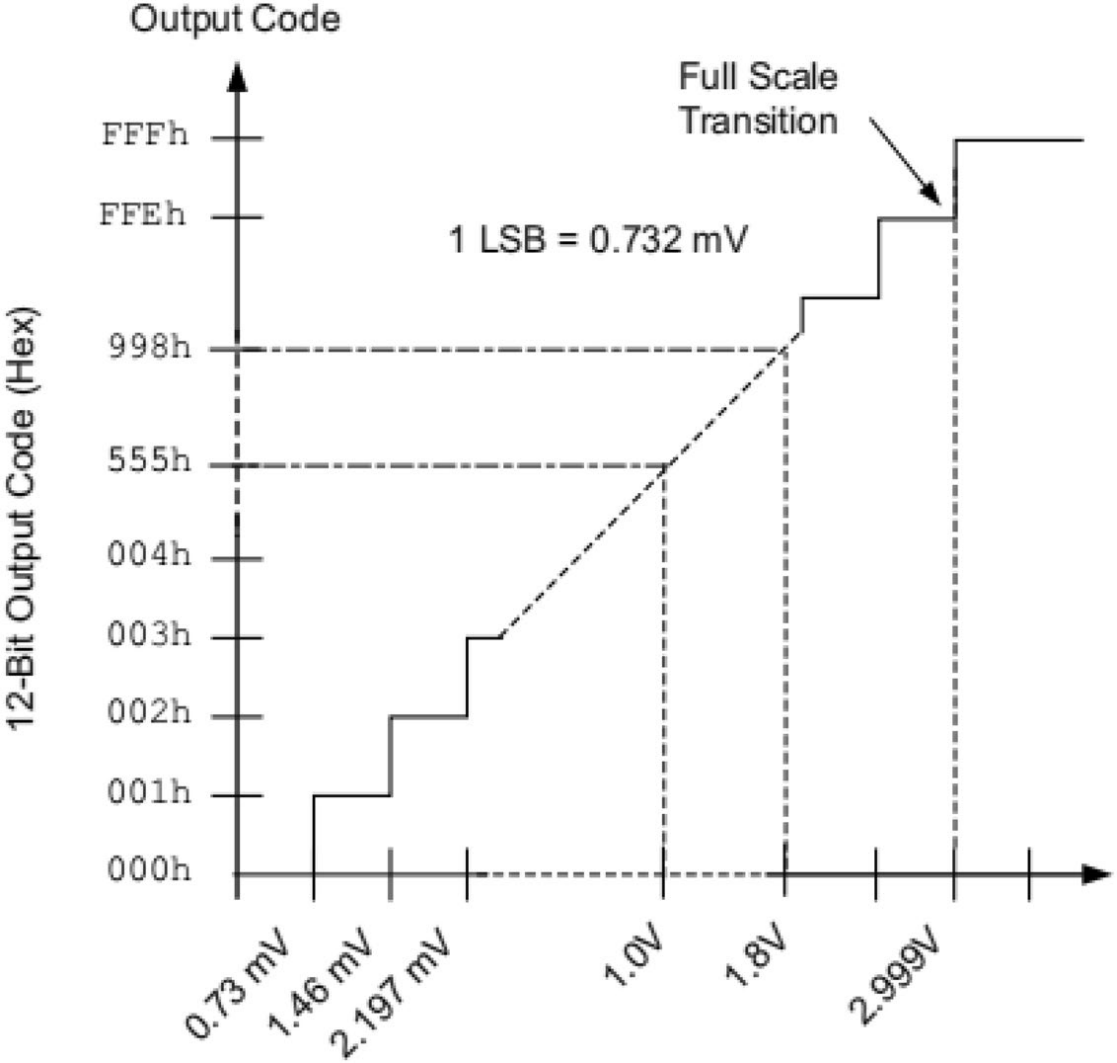
ZYNQ A/D data conversion diagram (taken from the datasheet for the UG480 (page 34)).

One of the ports is applied to acquiring external signals through the SMA interface input. After the signal is acquired, it is converted into differential signals within the XADC module of the FPGA, followed by data processing. The FPGA processing signal model is illustrated in Fig. 4. The voltage value is represented as a 16-bit binary value obtained from the output, and the relationship between the voltage value and the ADC code is given by the formula:

$${\text{Voltage Value}} = \frac{{ADC{ }Code}}{4096}{ } \times 3V;$$

Once the 16-bit number is obtained after the analog-to-digital (AD) conversion, it will be stored in RAM sequentially in accordance with its corresponding address. After the exposure is completed, the data stored in RAM will be sent to the host computer in the order of the address. Upon receiving the transmission instruction from the host computer, the AD-converted data will be processed in the host computer. By applying the voltage calculation formula to the received data, the voltage value of the corresponding pixel can be determined.

### Data transmission circuit

**Figure 5:**
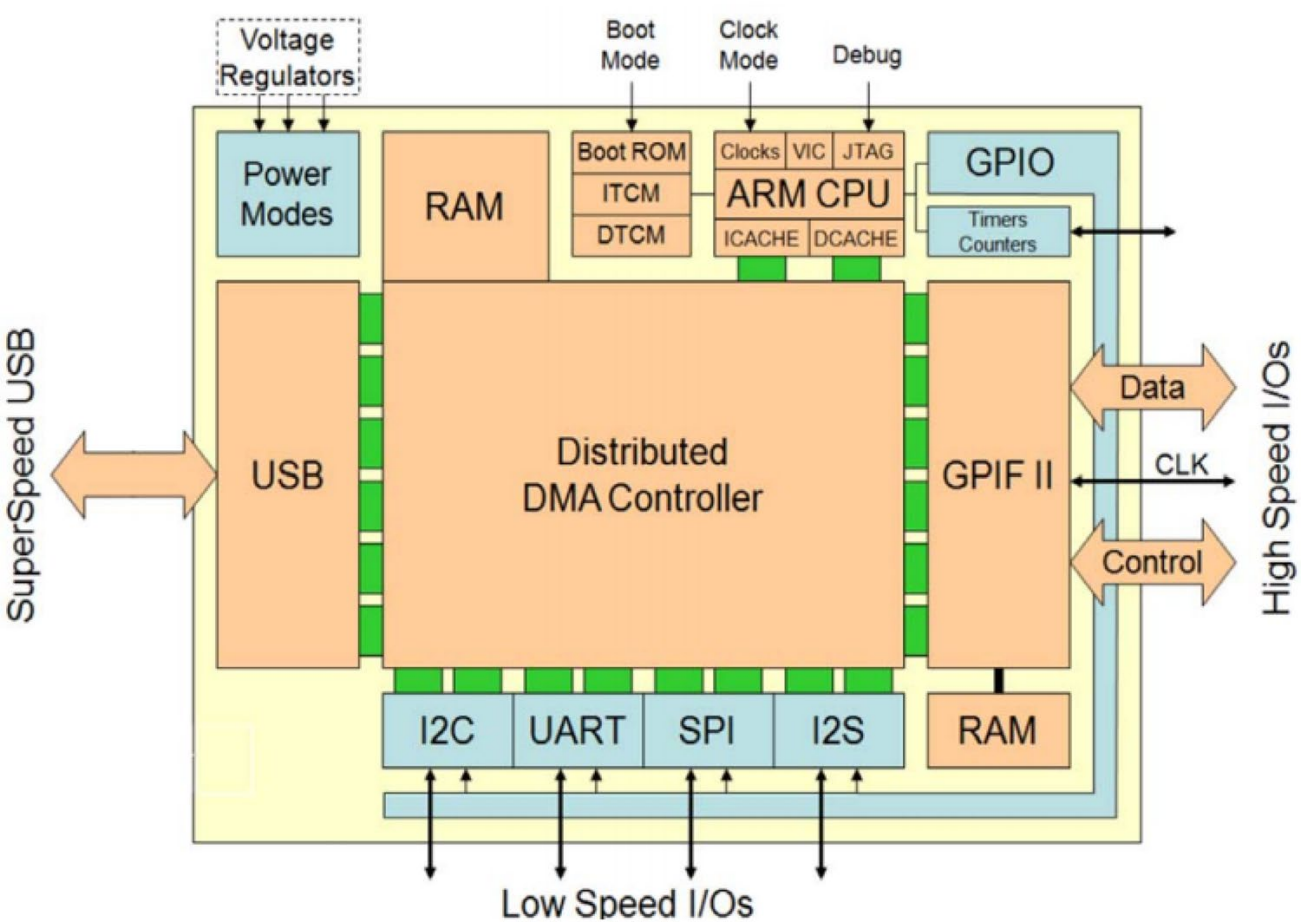
CYUSB3KIT-003 logic block diagram (taken from the datasheet for the CYUSB3KIT-003 chip (page 3)).

The FPGA needs to transmit the CCD signal data to the host computer for better visualization and analysis of the collected data. For this purpose, the data transmission module is employed, which is based on Cypress’s CYUSB3KIT-003 development board. The logical block diagram of the board is depicted in Fig. 5. The board’s functionality conforms to the USB 3.1 specification version 1.0 (TID # 340800007), meeting the requirements for USB 3.1 Gen 1 and USB 2.0. To facilitate data transfer, the programmable interface (GPIF II) of the development board is controlled by the FPGA. This allows for the utilization of 8-, 16-, 24-, and 32-bit data buses for the data transfer process.

## System software design

Based on the introduction of Chapter 3, the overall software design can be categorized into four modules: driver module, data processing module, data transmission module, and host computer module. These modules fulfill different functions in the system. The driver module is responsible for controlling the TCD1304DG chip.

It provides the necessary commands and instructions to configure and operate the chip effectively. The data processing module primarily focuses on converting the signals collected by the TCD1304DG into analog-to-digital (AD) format. The converted data is then stored in the FPGA’s RAM. At the same time, the module sends corresponding handshake instructions to the host computer for establishing synchronization. The data transmission module operates as follows: Upon receiving instructions from the host computer, it follows the integration time and the number of samples specified in the instructions. During this period, the host computer continuously accesses the FPGA’s working state.

**Figure 6:**
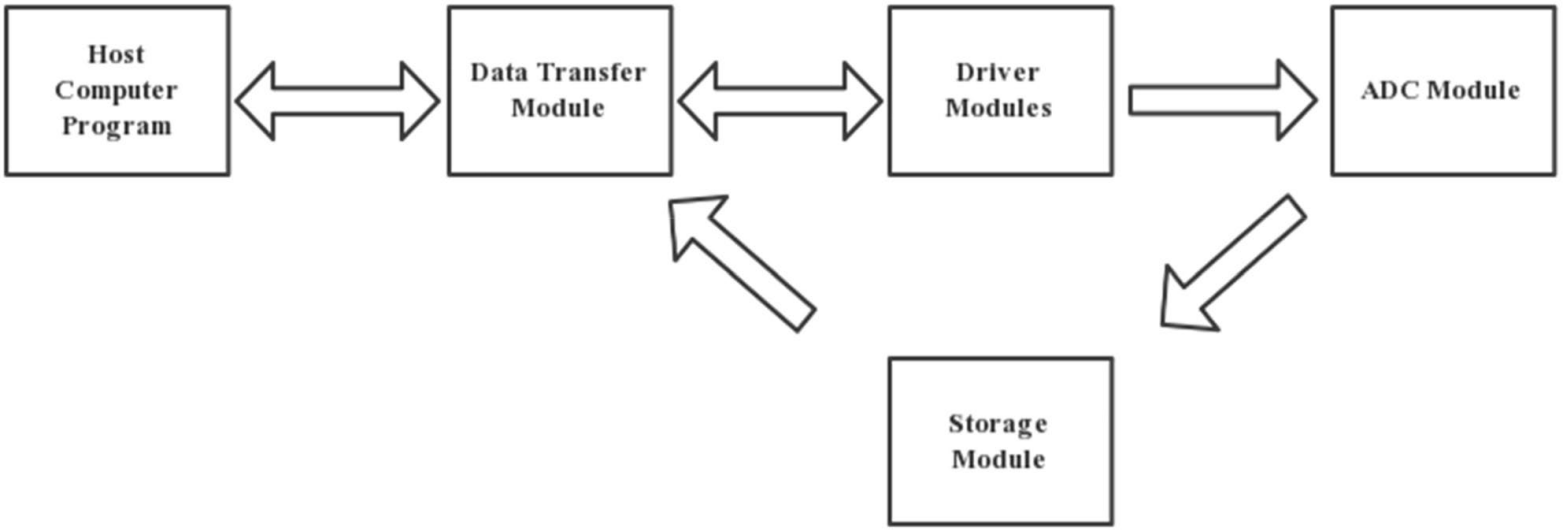
General design block diagram.

The module ensures that the data transfer aligns with the specified integration time and sampling requirements. The data transfer module functions as follows: After receiving instructions from the host computer, it processes the integration time and sampling times specified in the instructions. During the working period, the host computer continuously monitors the FPGA’s working state. Once the CCD exposure time is completed, the module sends an end instruction to the host computer. Subsequently, the host computer can send a receive data instruction to the FPGA. Upon receiving the corresponding instruction, the FPGA transfers the data stored in the RAM to the host computer based on the provided address. The data which is processed on the host computer side is carried out according to the voltage calculation formula described in Chapter 3. The host computer performs calculations and further processing on the received data on the basis of the voltage calculation formula outlined in Chapter 3. Among these modules, the first three are compiled by the Verilog programming language, while the host computer module is implemented by Python. Figure 6 illustrates the overall design block diagram, providing an overview of the system’s components and their interconnections.

### Driver module

There are three control pins of the TCD1304DG: ${V}_{SH}$, ${V}_{ICG}$, and ${V}_{\varphi M}$. As discussed in Chapter 3, ${V}_{\varphi M}$ serves as the fundamental master clock signal for the entire system, ${V}_{ICG}$ is responsible for charge reset, and ${V}_{SH}$ controls the integration time. The integration time duration is determined by the time interval between two falling edges of ${V}_{SH}$ when ${V}_{ICG}$ is at a low level.

**Figure 7:**
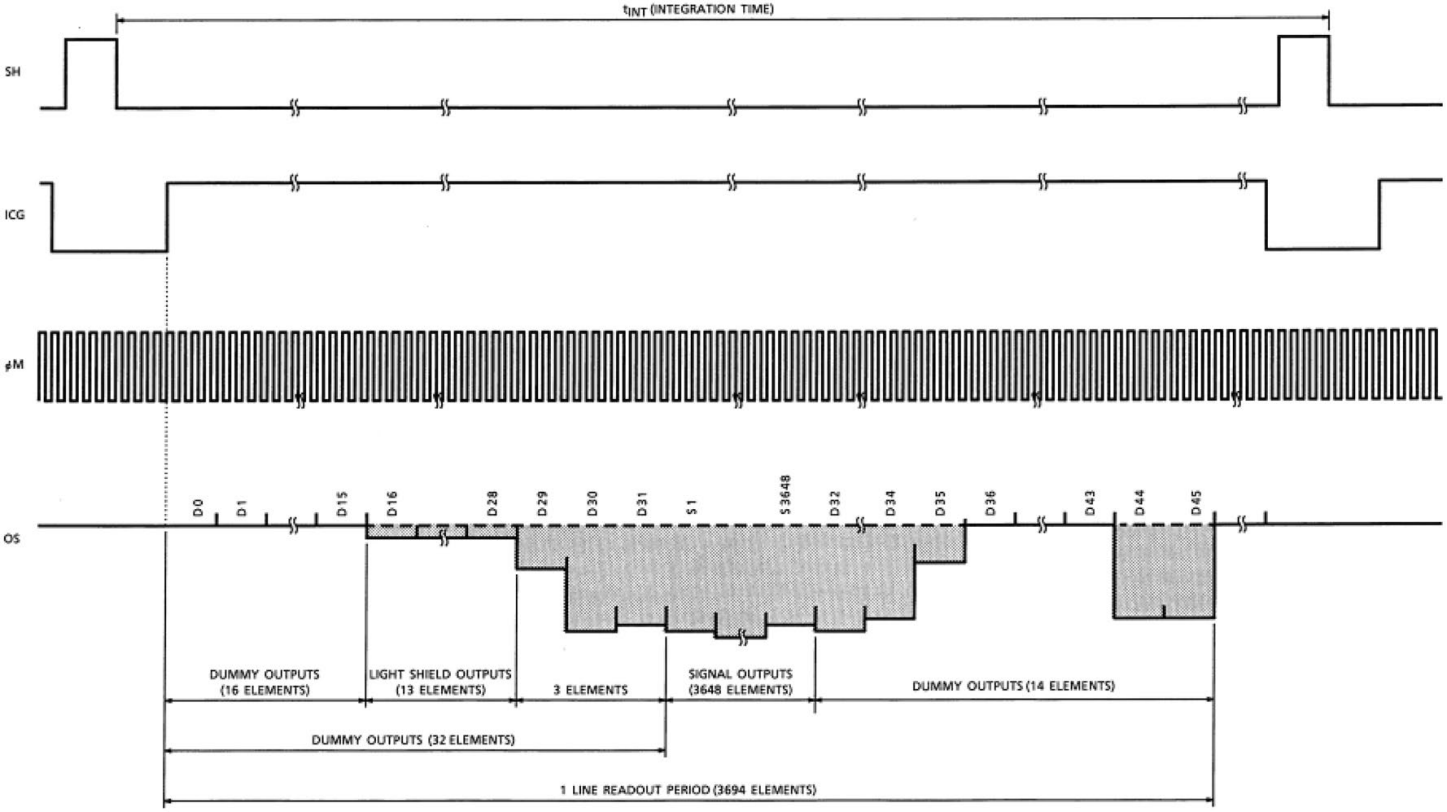
TCD1304DG timing diagram (taken from the datasheet for the TCD1304DG (page 6)).

The output time of the TCD1304DG refers to the time which takes for the CCD to output data values corresponding to all the pixels in the array. The key aspect of this design is controlling the CCD’s acquisition process by managing the timing of ${V}_{SH}$, ${V}_{ICG}$, and ${V}_{\varphi M}$. The specific timing diagram can be found in Fig. 7.

**Figure 8:**
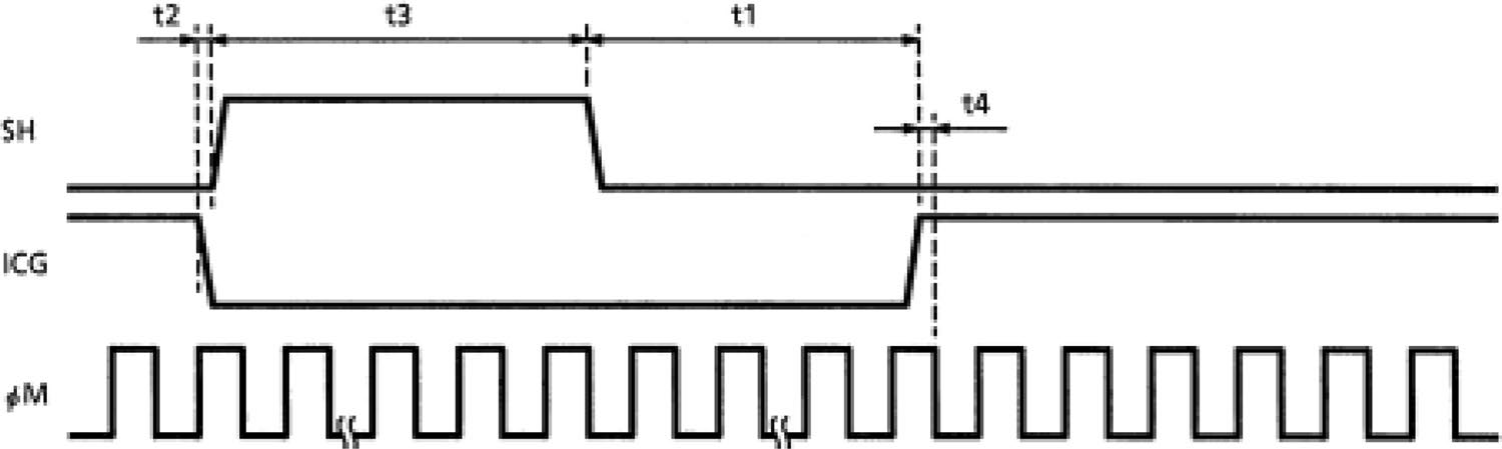
Control timing diagram (taken from the datasheet for the TCD1304DG (page 8)).

According to the requirements of the official data sheet, the timing difference between ${V}_{SH}$, ${V}_{ICG}$, and ${V}_{\varphi M}$ has strict requirements, and once it exceeds the requirements, the TCD1304DG will cut off the output, so the constraints of these three in the process of designing must be strictly referenced to the requirements in the data sheet, and the specifics are shown in Table 1 and Fig. 8.

**Table 1:** Control timing chart.

Characteristic	Symbol	Min (ns)	Max (ns)
${V}_{ICG}$ pulse delay	t1	1000	–
Pulse timing of ${V}_{SH}$ and ${V}_{ICG}$	t2	100	1000
${V}_{SH}$ pulse width	t3	1000	–
Pulse timing of ${V}_{ICG}$ and ${V}_{\varphi M}$	T4	0	–

In this design, the ${V}_{\varphi M}$ period is set to 2 MHz. The time parameters are as follows: t1 is 5000 ns, t2 is 500 ns, and t3 is 2500 ns. Based on these values, we can determine that the low pulse time of V_ICG in a single pulse is 8000 ns. Additionally, each pixel data output requires 4 cycles of ${V}_{\varphi M}$. Therefore, the total period of V_ICG can be calculated as follows:

$${3648}*\left( {{5}00*{4}} \right)\, + \,{8}000\, = \,{7},{3}0{4},000 {\text{ns}}.$$

**Figure 9:**
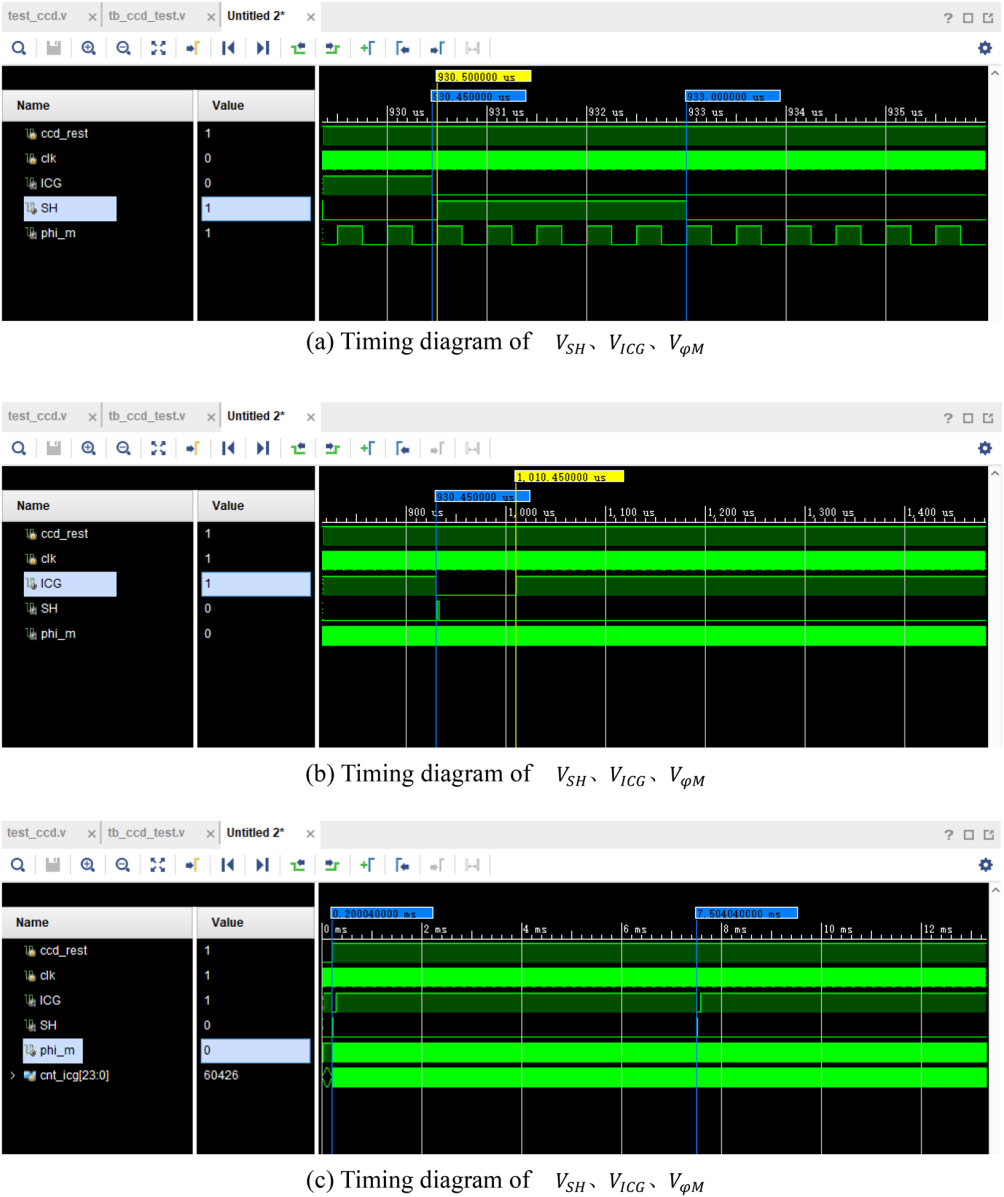
(**a**) Timing diagram of ${V}_{SH}$, ${V}_{ICG}$, ${V}_{\varphi M}$. (**b**) Timing diagram of ${V}_{SH}$, ${V}_{ICG}$, ${V}_{\varphi M}$. (**c**) Timing diagram of ${V}_{SH}$, ${V}_{ICG}$, ${V}_{\varphi M}$.

Therefore, in the design of ${V}_{SH}$, ${V}_{ICG}$ and ${V}_{\varphi M}$, it is possible to use one counter for the first two signals, while a separate counter is used for ${V}_{\varphi M}$ to maintain code simplicity. The ${V}_{SH}$ signal lags behind ${V}_{ICG}$ by 500 ns, and their respective pulse widths are set according to the provided table. However, the period of both signals is the same, which is 7.304 ms. As for V_φM, it was previously set to 2 MHz, corresponding to a period of 500 ns. The resulting timing diagrams for ${V}_{SH}$, ${V}_{ICG}$ and ${V}_{\varphi M}$ are depicted in Fig. 9a–c.

From Fig. 9a–c, it is evident that the timings of t1, t2, t3, and t4 between ${V}_{SH}$, ${V}_{ICG}$, and ${V}_{\varphi M}$ align precisely with the expected set values.

### Data processing modules

The main function of the module is to transmit the analog voltage value from the TCD1304DG to the FPGA’s integrated XADC (XILINX Analog-to-Digital Converter) for analog-to-digital conversion. This is achieved through the XADC input interface on the AXC7Z010 expansion board. During the analog-to-digital conversion process, it is important to consider the maximum sampling rate of the ADC module. This limit determines the maximum speed at which the ADC can perform conversions^18^. According to Nyquist’s Law of Sampling, the frequency of the sampled signal cannot exceed half of the ADC’s sampling rate when using the ADC for data sampling^19^. From the previous section, it was determined that the TCD1304DG takes four ${V}_{\varphi M}$ cycles to output the data value of a single pixel, with a ${V}_{\varphi M}$ frequency of 2 MHz. Therefore, the dual 12-bit 1 MSPS (Mega Samples Per Second) analog-to-digital converters available in the 7-series FPGAs and Zynq-7000’s SOC XADC can be utilized for the data conversion of TCD1304DG, given a master clock ${V}_{\varphi M}$ of 2 MHz.

**Figure 10:**
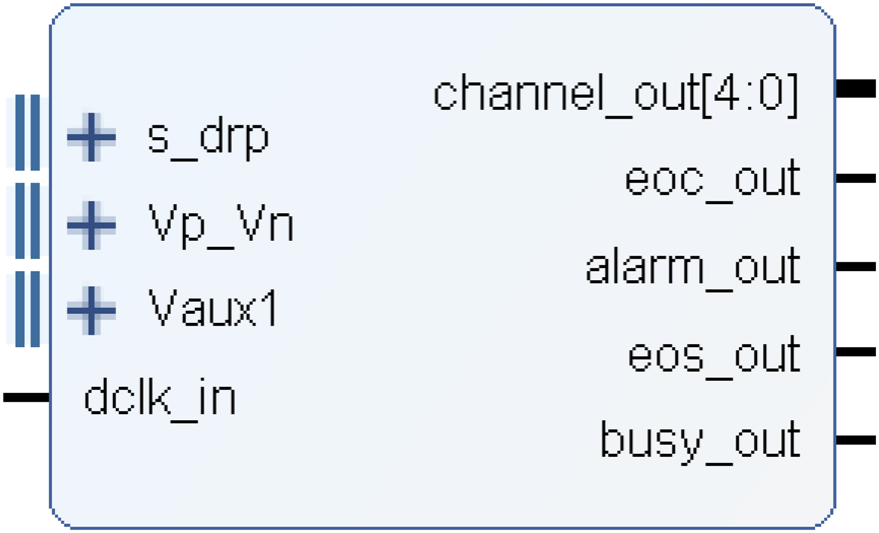
XADC IP core interface.

The use of this 7 series XADC in Verilog requires the use of an official IP core, which can be used after a simple configuration of the XADC. The IP core interface schematic for the XADC is shown in Fig. 10.

**Figure 11:**
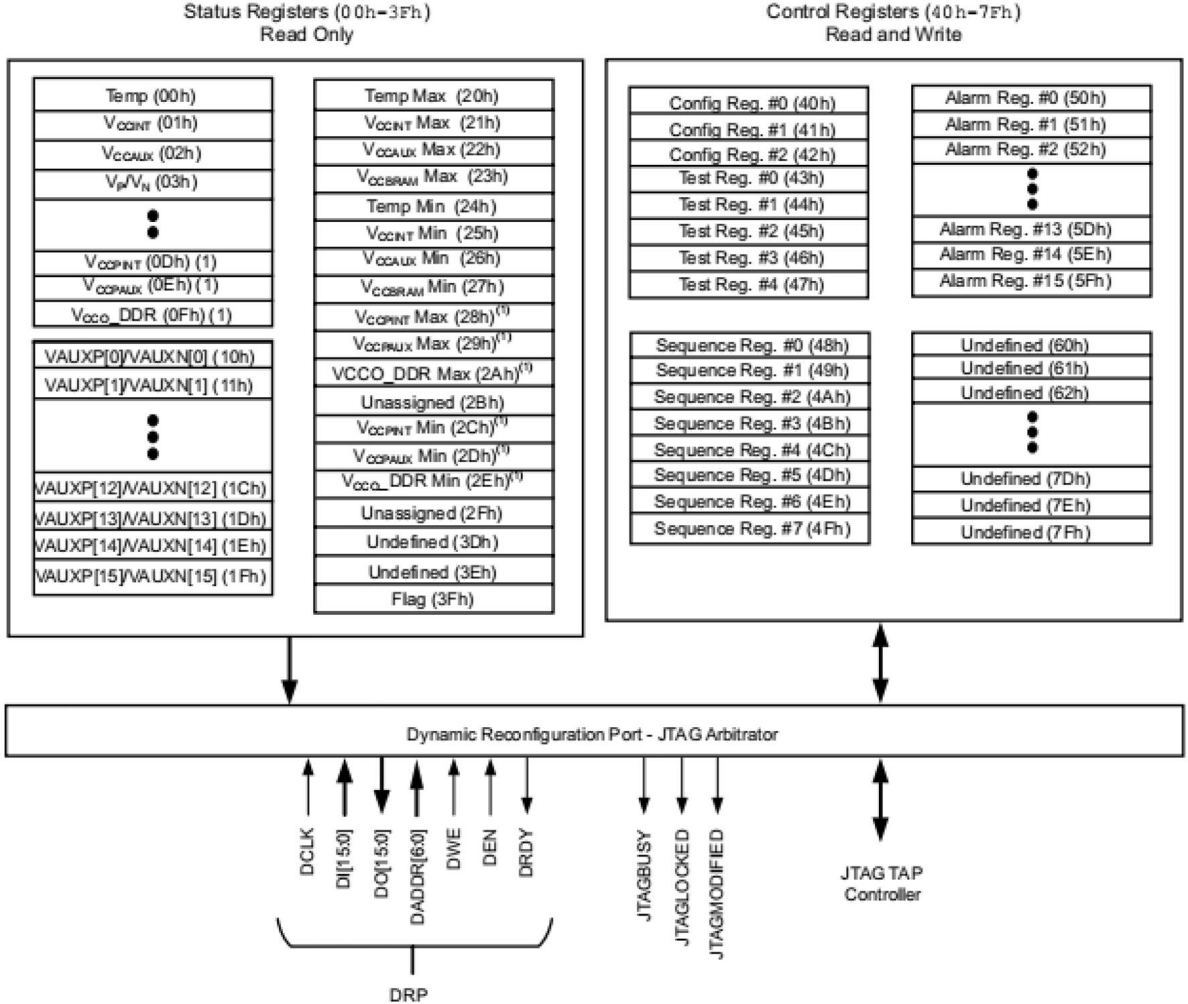
XADC register address (taken from the datasheet for the UG480 (page 36)).

It is important to note the interface "s_drp" in the "daddr_in" when working with the XADC. This interface is responsible for converting the channel address. Each different address corresponds to different conversion data. The specific registers corresponding to each address are depicted in Fig. 11.

According to the register address corresponding to the analog signal access channel and setting the appropriate enable signals for conversion, a 16-bit data can be obtained. This data represents the current analog signal converted by the XADC. The upper 12 bits of the data contain the conversion result, which is the most significant part. The lower 4 bits of the data, known as the unquoted data, can be used for techniques such as quantization or filtering, so as to enhance the resolution.

### Data transfer module

**Figure 12:**
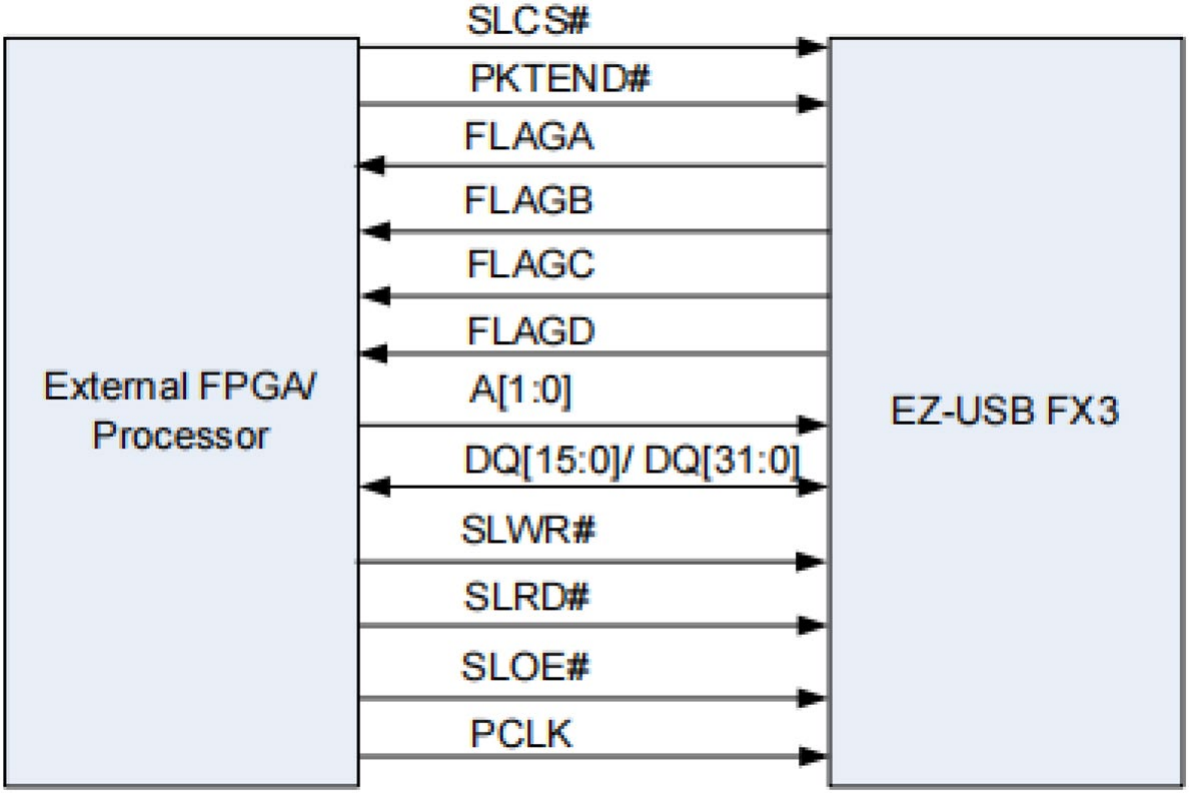
Synchronized slave device interface diagram (taken from the datasheet for the Infineon-AN65974 (page8)).

The data transfer module in this design utilizes Cypress’s CYUSB3KIT-003 for high-speed USB 3.0 transmission. For instance, in Si Yong Fu’s work^20^, USB2.0 was used for the TCD1304DG acquisition work. However, it’s worth noting that USB3.0 (480 Mbps) offers significantly faster data transmission compared to USB2.0 (4.8 Gbps). This increased speed in CCD data transmission can have a substantial impact on reducing the time-consuming burden of data processing by the host computer software, particularly in spectrometer design. The control mode is implemented through Cypress’s programmable interface called GPIF II, which offers various operating modes. In this design, the FIFO slave device operating mode is adopted. The schematic of the synchronous slave device interface is depicted in Fig. 12.

**Figure 13:**
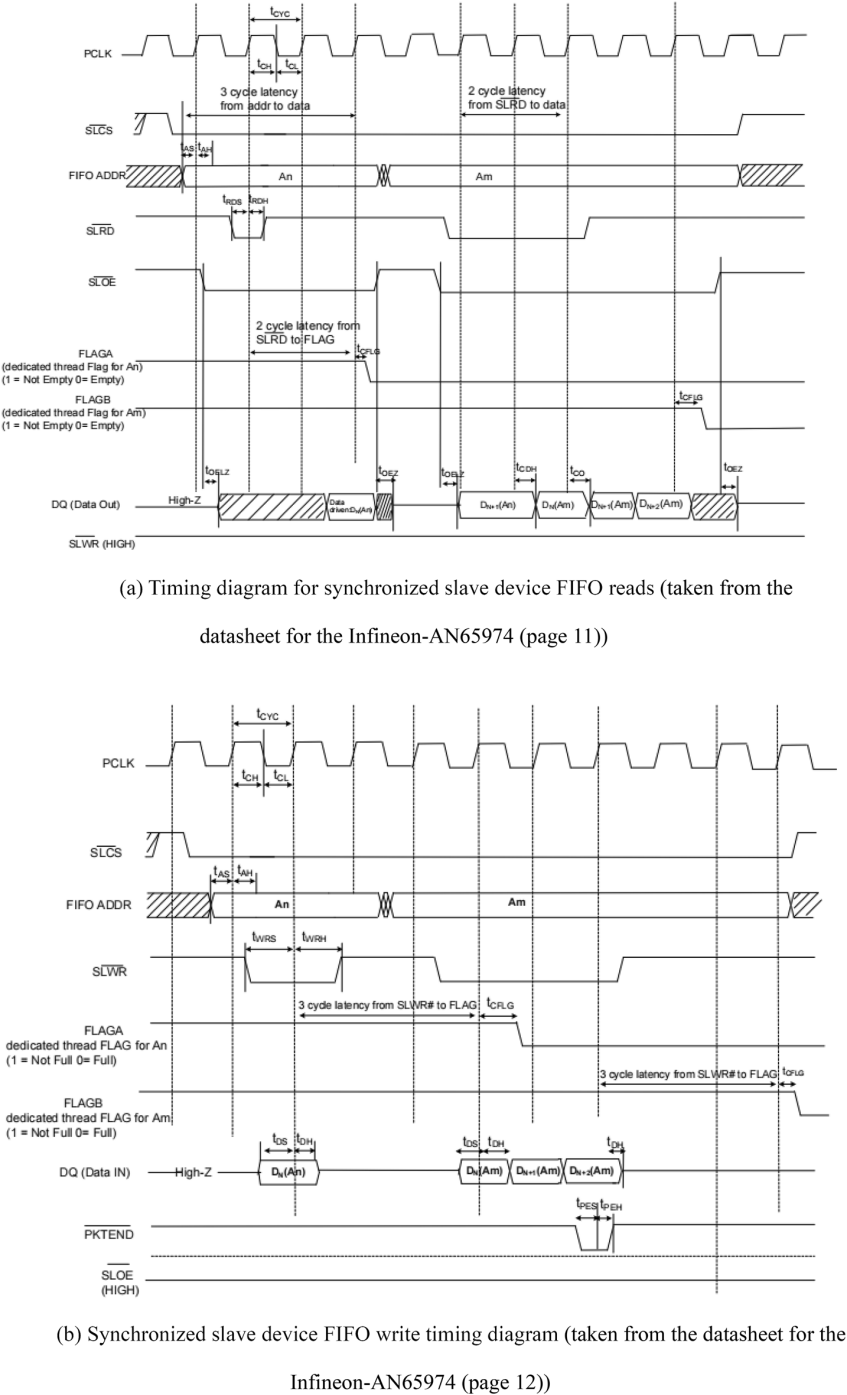
(**a**) Timing diagram for synchronized slave device FIFO reads (taken from the datasheet for the Infineon-AN65974 (page 11)). (**b**) Synchronized slave device FIFO write timing diagram (taken from the datasheet for the Infineon-AN65974 (page 12)).

The interface of CYUSB3KIT-003 is controlled by FPGA for data transfer purpose, and its synchronous slave device FIFO read timing diagram as well as write timing diagram are shown in Fig. 13a,b;

When reading data from the FPGA to the CYUSB3KIT-003, it is necessary to stabilize the FIFO address first. Then, the SLCS and SLOE signals should be pulled down. It is important to note that SLOE is an output enable signal, and when it is high, no operation on the data bus will be executed. After that, the SLRD signal should be pulled down, allowing the FPGA to read the data transmitted through the CYUSB3KIT-003 data bus.

When writing data from the FPGA to the CYUSB3KIT-003, it is crucial to specify the FIFO address and pull down the SLCS signal. Subsequently, the SLWR signal should be pulled down, enabling the internal FIFO of the CYUSB3KIT-003 to sequentially store the data from the data bus with the assistance of the PCLK clock signal.

**Figure 14:**
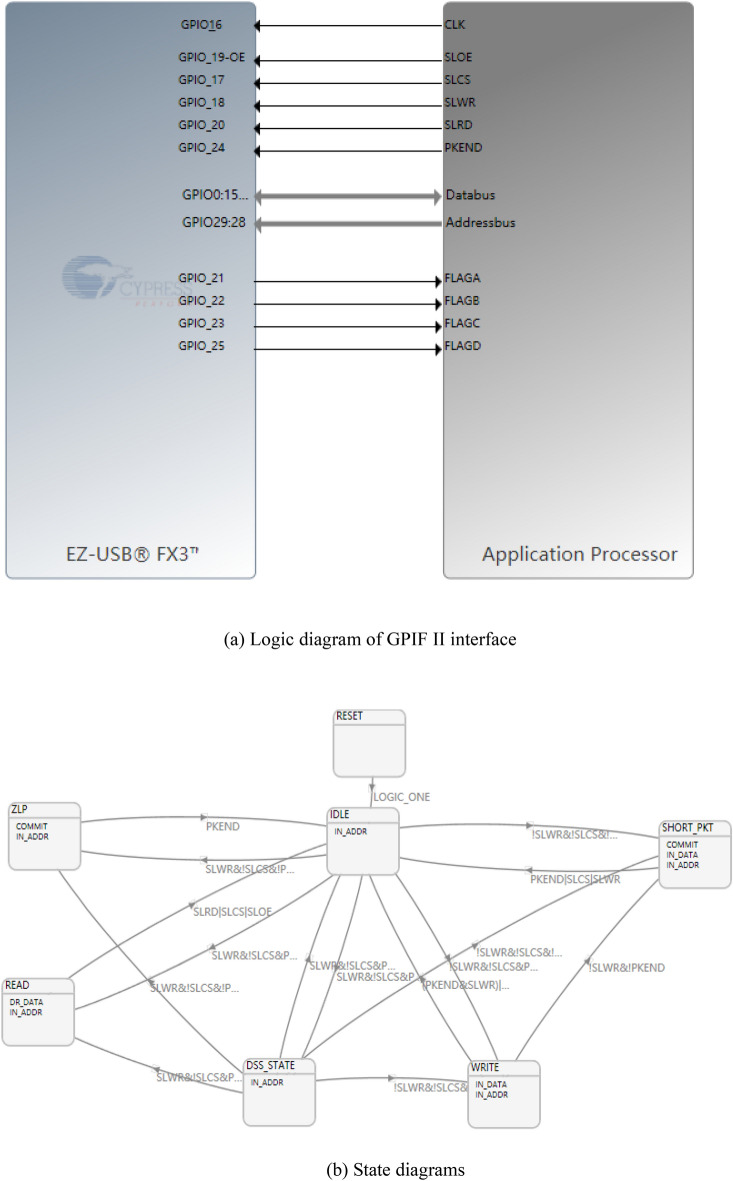
(**a**) Logic diagram of GPIF II interface. (**b**) State diagrams.

Concerning the CYUSB3KIT-003, it is important to note its multifunctionality, allowing it to serve as both a master and slave device. Therefore, in the design, it requires programming of its internal ARM and GPIF II interfaces. The logic diagram and state diagram of its GPIF II interface are displayed in Fig. 14a,b respectively. In this work, the PCLK was set as 100 MHz.

**Figure 15:**
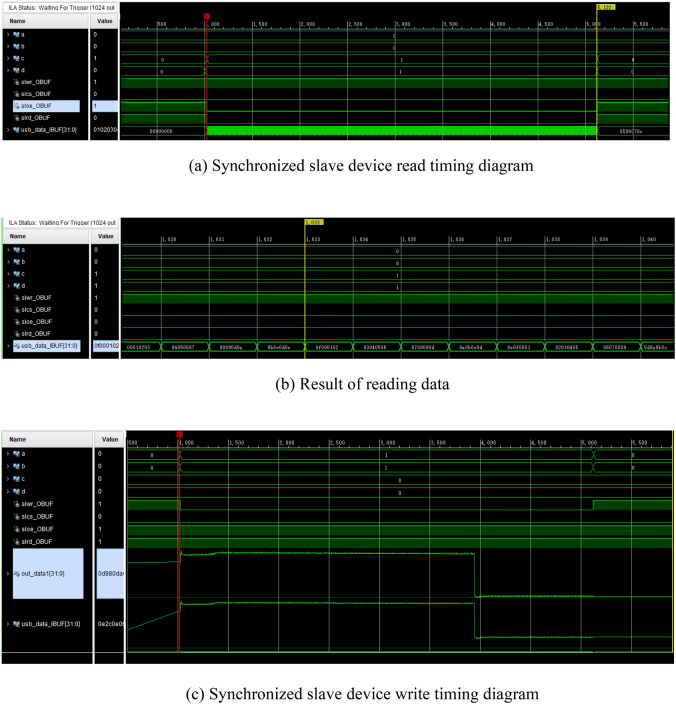
(**a**) Synchronized slave device read timing diagram. (**b**) Result of reading data. (**c**) Synchronized slave device write timing diagram.

Based on the GPIF II interface logic diagram and the synchronous slave device read/write timing diagram for FPGA programming, the timing design of each corresponding IO port signal yields the results presented in Fig. 15a–c.

To ensure accurate verification of the transmission process, a specific approach is employed. When the FPGA reads data from the CYUSB3KIT-003, the CYUSB3KIT-003 transmits the data to the FPGA based on the 00—0F cycle, Fig. 15b illustrates the captured data of the USB module with the FPGA in the logic analyzer, demonstrating that SLCS, SLOE, SLRD are pull down, while flag_c and flag_d are pull up after the data in accordance with the 00-0F cycle, the observed result aligns with the expected outcome. However, when the FPGA writes data to the CYUSB3KIT-003, a register named "out_data1" is configured in the FPGA to store the data. This register is then connected to the data bus of the CYUSB3KIT-003. Figure 15c depicts the change in the "usb_data" value with respect to "out_data1" after SLWR is pulled low and flag_a and flag_b are pulled high. This process demonstrates that the transmitted data matches the data of the FPGA after confirming the completeness and accuracy of the timing and logic interfaces between the FPGA and CYUSB3KIT-003.

## Experimental results

After completing the merging of all sub-modules, including the top-level module, the integrity of the entire design began to test. Started from placing the TCD1304DG under a light source. The output of the TCD1304DG was connected to the input of the oscilloscope.

**Figure 16:**
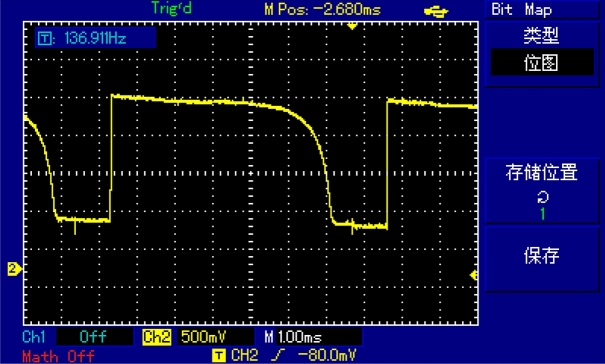
Oscilloscope display.

Next, the TCD1304DG was placed in a shady environment, and use black adhesive tape to cover three-quarters of it. From a distance of 10 cm, the TCD1304DG was illuminated vertically by a light source. The waveforms are observed and recorded under these conditions. The results of the testing process, along with the recorded waveforms, can be found in Fig. 16.

From the waveforms, it is evident that the output of the TCD1304DG exhibits a stepped pattern. This behavior can be attributed to the fact that only one-fourth of the sensor is illuminated by the light source. Consequently, the output is changed from a value close to three-fourths of its full range.

**Figure 17:**
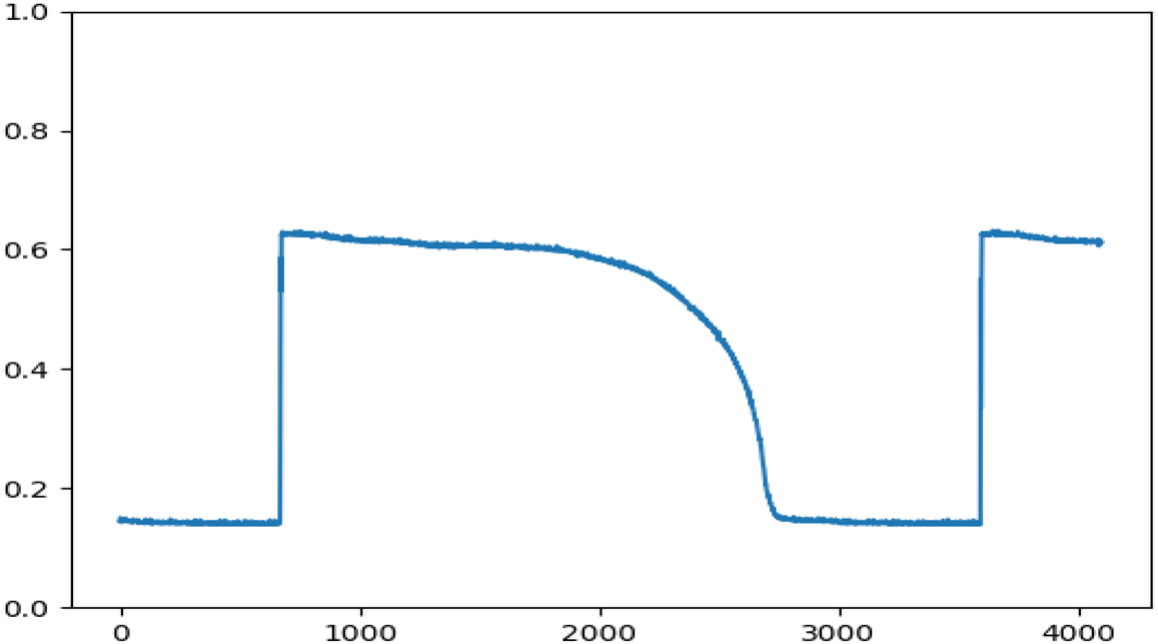
Upper computer data graph.

Following the normal measurement of the TCD1304DG’s output, it is connected to the AD input on the FPGA expansion board to verify the expected data that is displayed on the host computer. The upper computer program, coded in Python, utilizes the official USB driver library provided by Cypress to establish the USB driver for the host computer. This program receives data transmitted from the FPGA and converts each pair of data bits into the corresponding voltage value. The data is then graphed on the window interface using the matplotlib library. The results of this process are illustrated in Fig. 17.

By comparing Figs. 16 and 17, it becomes evident that the waveforms displayed in both figures are consistent. This observation serves as the evidence for the integrity of the XADC analog-to-digital converter module, the serial transmitter module, and the timing control of the TCD1304DG, the accuracy of data reading as well.

**Figure 18:**
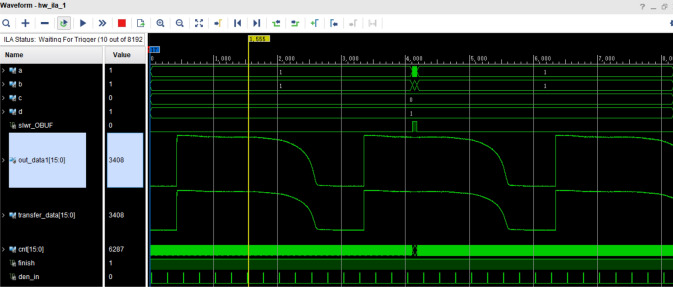
Logic analyzer timing diagram.

Figure 18 depicts the captured packet data from each model of the ILA logic analyzer during the process of data transmission. In this figure, "out_data1" represents the data which is read from the RAM, "transfer_data" corresponds to the transmitted data, and the signals A, B, C, and D represent the FLAG flag bits of the CYUSB3KIT-003 interface. The analog outputs of the TCD1304DG are compared with the analog outputs of the TCD1304DG through the AD converter. The resulting analog outputs are then transmitted to the upper computer for display. The comparison and transmission process validate the completeness and accuracy of the data, as demonstrated by Fig. 16 (the waveform detected by oscilloscope), Fig. 17 (the data received and plotted by the host computer), and Fig. 18 (the data extracted from the RAM on the FPGA side and the data transmitted by the USB module).

## Concluding remarks

Through a series of module designs, this project provides a comprehensive description of how to efficiently and cost-effectively utilize the TCD1304DG for optical signal data processing. The primary control is accomplished through an FPGA chip, which, in comparison to traditional MCUs, eliminates the need for CPU data processing and can be understood as a targeted hardware circuit design. This characteristic grants FPGA the advantages of high flexibility, high performance, and low power consumption. Furthermore, efficient data interaction is facilitated by employing high-speed USB 3.0 data transmission, enabling a higher data transmission limit for subsequent scalable functions. With this working design, the data processing process of the spectrometer becomes more efficient and stable.

The results of this design are particularly valuable for the spectral data acquisition process in the spectrometer, as they enable the acquisition of accurate and reliable output results after wavelength calibration of the spectral data. The theoretical expectations of this design have been successfully validated, demonstrating the correctness and feasibility of the proposed approach. This work could be carried out in a variety of applications that require sensor control and data transmission. For example, relocating FPGA and USB 3.0 into Masud Usman’s work for sensors might result in better detection efficiency, which would be a good direction for research^21,22^.

## Data Availability

The data that support the findings of this study are available from the corresponding author upon reasonable request. The test.bin file in the additional file is the CCD raw data read by the host computer from the RAM of the FPGA as shown in Chapter 5, and the data1.txt file is the CCD raw data converted from the analog voltage value in Section "Signal processing circuit" of the article.

## Abbreviations

AD: Analog-to-digital
ADC: Analog-to-digital converter
XADC: XILIN analog-to-digital converter
FPGA: Field programmable gate array
MCU: Microcontroller unit
CPU: Central process unit
